# Postmortem distribution of MDPHP in a fatal intoxication case

**DOI:** 10.1093/jat/bkae092

**Published:** 2024-11-28

**Authors:** Emma Beatrice Croce, Alexandra Dimitrova, Maria Grazia Di Milia, Stefano Pierotti, Davide Arillotta, Marta Barbaresi, Martina Focardi, Fabio Vaiano

**Affiliations:** FT-LAB, Department of Health Science, University of Florence, Florence, 50134, Italy; FT-LAB, Department of Health Science, University of Florence, Florence, 50134, Italy; FT-LAB, Department of Health Science, University of Florence, Florence, 50134, Italy; Forensic Pathology Unit, Careggi University Hospital, Florence, 50134, Italy; Department of Neurosciences, Psychology, Drug Research and Child Health, Section of Pharmacology and Toxicology, University of Florence, Florence, 50134, Italy; Department of Medicine and Surgery, Unit of Legal Medicine, University of Parma, Parma 43100, Italy; Department of Chemistry, Life Sciences and Environmental Sustainability, University of Parma, Parma 43121, Italy; Forensic Pathology Unit, Careggi University Hospital, Florence, 50134, Italy; FT-LAB, Department of Health Science, University of Florence, Florence, 50134, Italy

## Abstract

The synthetic cathinone (SC) 3,4-methylenedioxy-α-pyrrolidinohexanophenone (MDPHP) is structurally correlated to the 3,4-methylenedioxypyrovalerone (MDPV). In recent years, the number of intoxication cases has increased even if little is known about the pharmacokinetics properties. The Postmortem (PM) distribution of MDPHP remains largely unexplored. In these reports, MDPHP levels were quantified in blood, gastric content, and urine. This study aimed to describe the MDPHP PM distribution in several specimens, i.e. central and peripheral blood (CB and PB), right and left vitreous humor (rVH and lVH), gastric content (GCo), urine (U), and hair. The samples were collected from a cocaine-addicted 30-year-old man with a PM interval estimated in 3–4 h. Autopsy examination revealed unspecific findings, i.e. cerebral and pulmonary edema. No injection marks were observed. Toxicological analyses were performed using a multi-analytical approach: headspace gas chromatography for blood alcohol content (BAC), gas chromatography–mass spectrometry (GC–MS) for the main drugs of abuse, liquid chromatography–tandem mass spectrometry (LC–MS–MS) for benzodiazepines, and new psychoactive substances (NPS). BAC was negative (0.02 g/L). MDPHP concentrations were as follows: 1,639.99 ng/mL, CB; 1,601.90 ng/mL, PB; 12,954.13 ng/mL, U; 3,028.54 ng/mL, GCo; 1,846.45 ng/mL, rVH; 2,568.01 ng/mL, lVH; 152.38 (0.0–1.5 cm) and 451.33 (1.5–3.0 cm) ng/mg, hair. Moreover, hair segments were also positive for 3,4-dimethylmethcathinone (DMMC < limit of quantification: 0.01 ng/mg), α-PHP (0.59 ng/mg, 0.0–1.5 cm; 3.07 ng/mg, 1.5–3.0 cm), cocaine (6.58 ng/mg, 0.0–1.5 cm; 22.82 ng/mg, 1.5–3.0 cm), and benzoylecgonine (1.13 ng/mg, 0.0–1.5 cm; 4.30 ng/mg, 1.5–3.0 cm). MDPHP concentrations were significantly higher than those reported in the literature for fatal cases. For these reasons, the cause of death was probably the consumption of a lethal amount of MDPHP. Because CB and PB were similar, PM redistribution was not relevant.

## Introduction

Synthetic cathinones (SC) are a class of stimulants that belong to the category of New Psychoactive Substances (NPS). In Europe, more than half (>52%) of seized NPSs are SC, with five specific molecules accounting for nearly all SCs seizures: 3-chloromethcathinone (3-CMC), 3-methylmethcathinone (3-MMC), 4-chloromethcathinone (4-CMC), *N*-ethylhexedrone, and 3ʹ,4ʹ-methylenedioxy-α-pyrrolidinohexiophenone (MDPHP) [1]. These substances have contributed to the increase in both circulating NPS and fatal intoxication cases. In seven EU countries, the number of deaths involving SCs doubled in 1 year, from 14 in 2020 to 26 in 2021. Moreover, the EU’s early warning system has been alerted about a possible increase in SCs being mis-sold as MDMA or used as adulterants [1]. SCs act on the catecholaminergic system with sympathomimetic/amphetamine-like effects (such as euphoria, hyperactivity, sexual arousal, and cardiovascular risk) [2, 3]. On the other hand, SCs can also cause anxiety, insomnia, violent behavior, paranoia, arrhythmia, and hyperthermia. Substituents on the scaffold directly influence the pharmacodynamics and pharmacokinetics profiles. The presence of an *N*-pyrrolidine moiety induces a cocaine-like stimulation effect by inhibiting dopamine re-uptake [4]. One of the most representative *N*-pyrrolidine cathinones is methylenedioxypyrovalerone (MDPV), but many other compounds have been synthetized, such as MDPHP, α-pyrrolidinohexiophenone (α-PHP), and α-pyrrolidinopentiophenone e (α-PVP).

MDPHP is structurally correlated to MDPV, but structure–activity studies demonstrated that its longer aliphatic chain (4C vs. 3C for MDPV) increases the inhibition potency on the dopamine transporters [5]. MDPV and MDPHP are commonly inhaled (smoked or snorted). However, SCs reported modalities of intake also include oral consumption (i.e. “parachuting” or “bombing”), rectal (“boofing”), and intravenous (“slamming”) use [6, 7]. Little is known about the pharmacokinetic features of MDPHP because no studies are available; even less is known about Postmortem (PM) alterations. Although the number of reported cases of intoxication is increasing, deaths related to MDPHP use are extremely rare [8–13].

In this paper, a fatal intoxication case due to MDPHP was reported. The novelty of this study lies in the following: (1) the first quantification of MDPHP in vitreous humor and hair and (2) the first investigation of its PM redistribution.

## The case

A 30-year-old man was found dead in his bedroom, in supine position. The individual was last seen alive 24 h before being discovered. By the time of discovery, hypostasis and *rigor mortis* had already established.

The subject had a history of drug addiction, particularly chronic cocaine use.

## Materials and methods

### Chemicals and reagents

Dichloromethane (DCM), methanol (MeOH), hydrochloride acid (HCl), sodium hydroxide (NaOH), acetic acid, toluene, *N*-methyl-*N*-(trimethylsilyl)trifluoroacetaminde (MSTFA), ammonium hydroxide (NH_4_OH), *iso*-propanol (IPA), and acetonitrile (ACN) for sample treatment were obtained from Panreac Quimica S.L.U. (Castellar del Vallès, Spain). Water (H_2_O) and ACN for liquid chromatography–tandem mass spectrometry (LC–MS–MS) were acquired from Biosolve Chimie SARL (Dieuze, France). Formic acid was purchased from Merck KGaA (Darmstadt, Germany). Bond Elut LCR-certify 130 mg solid-phase extraction (SPE) cartridges were obtained from Agilent Technologies (Palo Alto, CA, USA). Methanolic standard solutions of MDPHP (0.02 mg/mL), α-PHP (0.02 mg/mL), and 3,4-dimethylmethcathinone (DMMC, 0.1 mg/mL) were purchased from Comedical s.r.l. (Trento, Italy) by the Italian Early Warning System and donated to our laboratory. Mephedrone-d_3_ (internal standard, IS; 0.1 mg/mL in MeOH) was supplied by LGC standards (Milan, Italy). All standards were then diluted to the appropriate concentrations in MeOH.

### Autoptic and histological analyses

A judicial autopsy was performed on the body, including a complete external and internal Postmortem examination. Organ samples, from the lungs, brain, heart, kidneys, spleen, pancreas, and liver, were included in paraffin, sectioned in micrometric slides using a microtome, stained with hematoxylin and eosin, and examined under a microscope. For toxicological analysis, central and peripheral blood (CB and PB), left and right vitreous humor (lVH and rVH), gastric content (GCo), urine (U), and hair samples were collected during the autopsy.

### Treatment of liquid samples for NPS analysis

A 200 µL aliquot of each liquid specimen (i.e. CB, PB, lVH, rVH, GCo, and U) was added with 700 µL of ACN (0°C) and 10 µL of IS (5 ng/µL). The mixture was then vortexed and centrifuged (2500 rpm, 5 min), and the supernatant was collected and dried under a nitrogen stream at 40°C. The residue was reconstituted with 100 µL of the mobile phase. Seven microliters was injected into the LC–MS–MS system. This method was previously validated: the limit of quantification (LOQ) for MDPHP was 0.5 ng/mL, with a recovery rate (RR) of 78.8% and an ion suppression (IoS) of −20.4% [14].

### Hair analysis

#### Sample pre-treatment

Two 1.5-cm segments (proximal 0–1.5 and 1.5–3.0 cm) of hair were analyzed. Before the extraction procedures, the samples were decontaminated twice with DCM (1 mL) and MeOH (1 mL), dried at room temperature, and cut into short pieces (<1 mm length) with scissors.

#### Main drugs of abuse

Hair samples (50 mg) were incubated at 45°C in HCl 0.1 M for 18 h, and then an SPE was achieved. SPE cartridges were preconditioned with 2 mL of MeOH and 2 mL of phosphate buffer (0.1 M, pH 6) and then washed with 2 mL of H_2_O, 1 mL of 1 M acetic acid and 2 mL of MeOH. The elution was carried out with 1 mL of mixture of DCM/IPA 8:2 (NH_4_OH 2%). The extract was dried under a nitrogen stream at 75°C. The residue was derivatized with 25 µL of MSTFA (75°C, 15 min) and then added with 25 µL of toluene. A 1 µL aliquot was injected into the gas chromatography–mass spectrometry (GC–MS) system [15, 16].

#### NPS

Hair samples (20 mg) were sonicated for 2 h at room temperature in presence of 1 mL of MeOH (formic acid 0.1%) and 10 µL of IS (1 ng/µL). The supernatant was collected and dried under a gentle nitrogen stream at 40°C. The residue was then reconstituted with 100 µL of the mobile phase. Seven microliters was analyzed by the LC–MS–MS system. MDPHP’s LOQ was estimated at 7 pg/mg, RR at 78.1%, and IoS at −21.3% [17].

#### Further analysis

Blood alcohol content (BAC) and the presence of the main drugs of abuse were investigated following previously published methods [16, 18]. In particular, BAC was measured using a head space (HS)-GC Agilent 7697A Headspace (Agilent Technologies). The biological fluids were treated following the same SPE procedure used for hair analysis. The GC–MS was an Agilent 7890A GC system equipped with an Agilent 7683B series autosampler (Agilent Technologies) and coupled to a single quadrupole Agilent 5975C mass spectrometer (Agilent Technologies). Chromatographic separation was performed using an Agilent HP-5 MS column (30 m length, 0.25 mm i.d. and 0.25 mm film thickness, Agilent Technologies). The gas carrier (He) flow was constant at 1 mL/min. The samples were analyzed with three different detection modes as previously published [16].

#### LC–MS–MS

LC–MS–MS instrument consisted of an HPLC Agilent 1290 Infinity system (Agilent Technologies) coupled to an Agilent 6460 Triple Quad MS (Agilent Technologies). Ionization by the electrospray ion source (ESI) was in positive mode. The ESI configuration was as follows: gas temperature at 325°C, gas flow rate at 10 L/min, nebulizer at 20 psi, and capillary at 4000 V. A reversed-phase C18 column was employed (Zorbax Eclipse Plus C18, 2.1 × 100 mm, 1.8 µm, Agilent Technologies). Gradient elution started from a mixture of 5 mM aqueous formic acid (A) and ACN (B) 99:1, increasing %B as follows: to 5% in 5 min; to 10% in 2 min and hold for 3 min; to 20% in 5 min; to 30% in 5 min and hold for 2 min; to 40% in 3 min; to 50% in 3 min; to 70% in 2 min; to 100% in 5 min and hold for 2 min; post-time fixed in 2 min. The flow rate was constant at 0.6 mL/min. The acquisition was performed in multiple reaction monitoring (MRM) mode. Transitions are reported in Table 1.

**Table 1.** MRM transitions and LC–MS–MS conditions

Compound	[M + H]^+^	Transitions (*m/z*)	Fragmentor (V)	CE (V)	RT (min)
MDPHP	290	**135**, 140	113	25, 29	15.6
α-PHP	246	**140**, 91	123	29,25	14.9
DMMC	192	**174**, 159	96	9, 21	11.7
Mephedrone-d_3_	181	**148**, 163	90	17, 5	7.6

CE: collision energy; RT: retention time.

Quantitative transitions are highlighted in bold.

## Results

### Autoptic examination and histological analysis

External examination showed no pathological or traumatic findings. No injection marks were observed. Macroscopic internal examination revealed pleurivisceral congestion and lung edema, confirmed by the histological examination. Moderate hepatic steatosis was also observed.

### Toxicological analyses

BAC was measured at 0.027 g/L by HS-GC. Biological fluids (CB, PB, U, GCo, rVH, and lVH) were negative to “common” drugs of abuse by GC–MS, while the general unknown screening (GUS) analysis provided a positivity for “4ʹ-methyl-a-pyrrolidinohexanophenone” with a likelihood of 72% (SWGDRUG MS Library Version 3.13). LC–MS–MS analysis revealed MDPHP as the only substance present in the liquid specimens as follows: 1,639.99 ng/mL, CB; 1,601.90 ng/mL, PB; 12,954.13 ng/mL, U; 3,028.54 ng/mL, GCo; 1,846.45 ng/mL, rVH; and 2,568.01 ng/mL, lVH (Table 2). To evaluate past consumption of any psychoactive substance, a hair sample (length 3 cm) was divided into two segments related to two different periods of consumption: 0.0–1.5 (last month, approximately) and 1.5–3.0 cm (second-to-third month before death, approximately). In addition to MDPHP, in both hair segments were also quantified cocaine (and its main metabolite, benzoylecgonine), α-PHP, and DMMC. The concentrations in the first and second segments were, respectively, 152.38 and 451.33 ng/mg for MDPHP; 0.59 and 3.07 ng/mg for α-PHP; <LOQ (0.01 ng/mg) for DMMC; 6.58 and 22.82 ng/mg for cocaine; and 1.13 and 4.30 ng/mg for benzoylecgonine.

**Table 2.** MDPHP concentrations in the biological samples and review of literature

Specimen	Concentrations (ng/mL or ng/mg)	Previously reported concentrations (ng/mL)					
		[8]	[9]	[10]	[11]	[12]	[13]
Central blood	1,639.99						
Peripheral blood	1,601.90	399	76	3–136	3.4	3.3–140	1.26–73.30
Urine	12,954.13	222		2–5960	30.5		19–8,769
Gastric content	3,028.54	50					
Vitreous humor (right)	1,846.45						
Vitreous humor (left)	2,568.01						
hair (0.0–1.5 cm)	152.38						
hair (1.5–3.0 cm)	451.33						
**Case**	Death	Death	Newborn death	Intoxication	Intoxication	Intoxication	Intoxication
**Other substances**	In hair: α-PHP, DMMC, cocaine	-	α-PHP	-	25E-NBOH	BDZ, COC, AMP, OPP, α-PHP^a^	COC, AMP, CAN, α-PHP
**Blood derivative**	Whole blood	Whole blood	Whole blood	Serum	Plasma	Serum	Whole blood

a Abbreviations: AMP, amphetamines; BDZ, benzodiazepines; CAN, cannabinoids; COC, cocaine; OPP, opiates/opioids.

## Discussion

The detection of NPS remains a challenge for forensic toxicologists, especially in PM analysis. In recent years, significant efforts have been made to overcome the lack of analytical standards and methods. In particular, an increasing number of screening procedures involving MS or HRMS have been developed to rapidly and sensitively identify a wide variety of molecules [19–22]. These wide-range analytical approaches can improve our understanding of the NPS phenomenon through their detection even when consumption is unsuspected. The reported case involved a 30-year-old man who had a well-known cocaine addiction. He was found dead in his bedroom, and autopsy examination revealed unspecific signs (pleurovisceral congestion and lung and brain edema). Adoption of a wide-range analytical strategy (GUS and LC–MS–MS screening) allowed us to quantify MDPHP at very high concentrations in all the biological fluids, thereby explaining the cause of death. In the literature, only one case of lethal intoxication by MDPHP in adults has been reported. Di Candia *et al*. reported the death of a 48-year-old man who was found in a semi-conscious state on a sidewalk. Initially, this subject exhibited a regular breathing rate and normal cardiovascular parameters. However, following hospitalization, he suddenly lost consciousness and died within minutes. MDPHP was quantified in PB, U, and GCo at 399, 222, and 50 ng/mL, respectively. According to the analytical evidence and circumstantial information, the authors supposed an oral intake 60–90 min before the first medical aid. Unfortunately, an accurate determination of the time of consumption was impossible due to the absence of witnesses or relevant circumstantial information. In addition, MDPHP concentrations were not helpful because pharmacokinetic properties are unknown; furthermore, in our case, we cannot exclude previous or multiple consumptions over a wide time range before the death (see high MDPHP level in U). PMI estimation was based on *rigor* and *livor mortis* in few hours. VH, CB, and PB levels were very similar; thus, we could exclude the onset of the PM redistribution; the higher lVH level could find an explanation in its partial coagulation. BAC was substantially negative (0.027 g/L); thus, alcohol did not contribute to the cause of death.

Hair analysis revealed past consumption of MDPHP, α-PHP, DMMC, and cocaine. MDPHP was detected in both hair segments, providing evidence of its use in the last 3 months. Since this is the first-ever case of MDPHP hair quantification, the rate of use/abuse cannot be estimated; however, concentrations were consistent with SC levels measured in other studies [23]. Hair analysis proved also the use of other SCs, α-PHP, and DMMC. In our opinion, their consumption was probably unintentional, likely due to their presence as adulterants or impurities of MDPHP. Indeed, concomitant use of MDPHP and α-PHP was already observed in a previous study in which the hospitalized subjects usually declared the use of “MDPV” or “PV”, and they were not aware that they were consuming a different SC [13]. Our conclusion may find further evidence in the low concentrations of DMMC and α-PHP: DMMC was barely detected (<LOQ in both hair segments), while α-PHP levels were lower than those quantified in hair samples from a continuous heavy consumer, i.e. 4,700.0 (0.0–2.5 cm) and 3,600.0 (2.5–5.0 cm) pg/mg [23]. Unfortunately, α-PHP hair concentrations are not available for “naïve” or “moderate” consumers. Our theory was also consistent with EMCDDA reports, in which SCs mis-sold or used as adulterants appear to be an increasing phenomenon [1].

In conclusion, MDPHP was quantified in VH and hair for the first time. Moreover, a comparison of its concentrations in PB, CB, and VH allowed us to obtain the first evaluation of its PM redistribution features.

## Data Availability

The data underlying this article will be shared on reasonable request to the corresponding author.
